# The Hygiene of the Mouth, a Guide to the Prevention and Control of Dental Diseases

**Published:** 1899-07

**Authors:** R. Dennison Pedley

**Affiliations:** Eng. Dental Surgeon to the Evelina Hospital for Sick Children, Southwark, London


### The Hygiene of the Mouth, a Guide to the Prevention and Control of Dental Diseases.

BY R. DENNISON PEDLEY, F.R.C.S., ED., L.D.S., ENG., DENTAL SURGEON TO THE EVELINA HOSPITAL FOR SICK CHILDREN, SOUTHWARK, LONDON.

This little work, just issued by the S. S. White Mfg. Co., of Philadelphia, is a work full of valuable suggestions to the students upon this branch of dental science.

Chapter 1 contains the hygiene of the mouth in children, in the home, in the school, in the hospital; the relationship between dental and other diseases.

Chapter 2 contains the hygiene of the mouth in adults, divided into signs of caries of teeth, and their results; exposed pulps; alveolar abscess; the loss of teeth; the effects upon other teeth; the effects of carious teeth upon the general health, and the methods of treatment; general summary.

The author starts out with a description of the diseases of childhood, presenting the various phases of affections to which they are subject, discussing each of these in respect to condition, etiology and treatment.

The study of prophylaxis is very clearly, though briefly, presented. The general truths in regard to these subjects are very well, though briefly, presented. Enough is given, however, to give the uninitiated good and valuable ideas in regard to the care of children's teeth.

The use of the brush with simple preparations as dentifrice, such as chalk, soap and kindred substances, is very strongly emphasized. The author lays much responsibility upon the dentist, urging that it should be an important part of his business to see, not only for the remedying of diseases, but for prevention as well, this to be done by impressing proper information upon the attention of mothers and, indeed, all having charge of children. He does not by any means excuse the physician from responsibility in this matter. He suggests the attention to the teeth of children in our public schools as very important.

The second chapter, "Oral Hygiene of Adults," is presented in its various aspects. And here, as with children, the great point presented is that of mouth cleanliness. The proper use of the teeth in mastication is strongly urged. In order to accomplish this, however, entire immunity from disease is necessary.

The work is well illustrated by quite a number of cases, illustrating faulty conditions of the teeth, both in respect to decay of the teeth, and the effect upon adjacent structures and irregularity.

The aim of the work seems to be to stimulate the reader to such care as will prevent the various affections that are all too common in the mouths of people, both young and old.

In conclusion, the author remarks that, while pointing out the measures to be adopted for the prevention of dental diseases in adult life, their importance has been emphasized by a brief description of dental caries, its progress, complications and treatment. The disadvantages of the loss of teeth and the subsequent effects upon other teeth have been considered.

The influence of neglected mouths upon the general health has been fully illustrated by individual cases. The cases chosen are typical of what may be found in the every-day experience of the dental surgeon.

This work is valuable and should be in the hands of every dentist, and every physician as well, and it would be vastly beneficial if placed within the reach of other intelligent people.

